# Understanding Sexual Activity and Chlamydia Testing Rate Based on Linked National Survey and Medicaid Claims Data

**DOI:** 10.1371/journal.pone.0122927

**Published:** 2015-04-13

**Authors:** Guoyu Tao, Jennifer Hua, Jessica L. Chen

**Affiliations:** Division of STD Prevention, National Center for HIV/AIDS, Viral Hepatitis, STD, and TB Prevention, Centers for Disease Control and Prevention, Atlanta, Georgia, United States of America; Leibniz Institute for Prevention Research and Epidemiology (BIPS), GERMANY

## Abstract

**Background:**

Monitoring adherence to national recommendations for annual chlamydia screening of female adolescents and young adult women is important for targeting quality improvement interventions to improve low screening rates. However, accurate measurement of rates may vary depending on the data source used to determine eligible sexually-active women.

**Methods:**

The 2001–2004 NHANES data linked with Medicaid administrative data by respondent’s unique identifier, the 2011–2012 NHANES data, and the 2004 and 2010 Medicaid data were used in this cross-sectional analysis. We defined self-reported sexual activity by self-reported sexual behaviors, claim-identified sexual activity by reproductive-related claims among women who had ≥ one healthcare claim, HEDIS-defined sexual activity by reproductive-related claims among women who were enrolled in Medicaid for ≥330 days and had ≥ one healthcare claim, and chlamydia tests by claims submitted in the 12 months prior to the survey interview.

**Results:**

Of Medicaid women aged 18–25 years, 91.5% self-reported to be sexually-active. Of self-reported sexually-active women aged 18–25 years, 92.0% had ≥ one healthcare claim in the 12 months prior to the survey interview; of this subpopulation, only 58.8% were enrolled in Medicaid for ≥ 330 days in the 12 months prior to the survey interview; of this further subpopulation, 74.1% had healthcare claims identifying them as sexually-active in the 12 months prior to the survey interview. Of HEDIS-defined sexually-active women, 42.4% had chlamydia testing.

**Conclusion:**

Our study suggests that the number of sexually-active women aged 18–25 years used as the denominator in the chlamydia testing measure could be significantly different, depending upon the definition applied and the data used. Our data highlight the limited representativeness of Medicaid population in the current HEDIS measure on chlamydia testing when a high proportion of women who were enrolled in Medicaid for <330 days had been excluded from the measure. The interventions that can improve the proportion of women who were enrolled in Medicaid for ≥ 330 days among all young Medicaid women are needed not only for improving health care services, but also for measuring quality of healthcare.

## Introduction

Chlamydial infection is the most commonly reported infectious disease, and its prevalence is the highest in women aged ≤ 25 years in the United States.[1] Because a high proportion of infected women are asymptomatic, healthcare providers frequently rely on screening to detect chlamydial infections. The Centers for Disease Control and Prevention (CDC) has recommended annual screening of all sexually-active women aged ≤ 25 years, or older women with risk factors (e.g., those who have a new sex partner or multiple sex partners).[2] Many other organizations have similar recommendations.[3–6] Sexual activity is normally identified by self-reported sexual behaviors, medical records, or healthcare claims.[7–9]

Efforts and activities to monitor chlamydia testing for sexually-active young women have been conducted in the United States. Using a national survey, chlamydia testing rates were estimated to be 43% in 2002, and 38% in 2006–2008 for all sexually-active U.S. women aged 15–25 years.[10,11] In 1999, the National Committee for Quality Assurance (NCQA), an independent, nonprofit organization that assesses and reports on the quality of care delivered by managed care organizations, instituted a performance measure for annual screening of sexually-active women aged 15–25 years for chlamydia into the performance measurement system known as the Healthcare Effectiveness Data and Information Set (HEDIS).[12] The HEDIS measure for chlamydia testing represents the continuously-enrolled and sexually-active women aged 16–25 years who were tested for chlamydia in the prior 12 months. Sexually-active women in the HEDIS measure were identified as women who had any reproductive-related medical or pharmacy claims. Continuously-enrolled women were those who had no more than a one-month gap if they were insured in Medicaid HMO plans. Monitoring of chlamydia testing trends based on the HEDIS data has shown consistent increases since the implementation in 2001, from 23.1% to 45.1% in 2012 among HMO commercial plans; and from 40.4% in 2001 to 57.1% in 2012 among Medicaid HMO plans.[13]

The aim of the current HEDIS measure on chlamydia testing is to minimize the number of false positives identified as part of the denominator population, and to make this denominator more closely reflect the at-risk population for chlamydial infection.[8] If self-reported sexual activity is known and considered to be the best indicator of whether a person is sexually-active, it can be used to identify four errors in the way of identifying sexual activity used in the HEDIS measure. One group of women will be erroneously included, and three other groups of women will be erroneously excluded. The inclusion group is the self-reported non-sexually-active women who were identified to be sexually-active by claims. The three exclusion groups are (1) the self-reported sexually-active women who had no medical or drug claims in the prior 12 months, (2) the self-reported sexually-active women who had a one-month gap in Medicaid coverage during a measurement year, and (3) the self-reported sexually-active women who had medical or drug claims, but these claims were not related to reproductive healthcare. Because these exclusions might result in an overestimated chlamydia testing rate (due to an artificially low denominator), many researchers have concerned about the measurement of sexual activity since this HEDIS measure was first used in 2000.[9,14–16] One previous study compared sexual activity measured by self-reported behavior using a national representative survey with that measured by healthcare claims using a large commercial claims database.[9] However, persons in one database could not be linked to persons in another database in that study. A recent study conducted in a commercial managed care organization is the only study so far that compared women’s sexual activity measured by self-reported behaviors to that measured by healthcare claims.[17] The study showed that of women aged 18–25 years who self-reported being sexually-active and who had no one-month gap in insurance coverage, approximately 85% were classified as sexually-active by the HEDIS measure; of women aged 18–25 years who self-reported being non-sexually-active, approximately 64% were classified as non-sexually-active by the HEDIS measure. To our knowledge, there are no nationally representative studies that have compared sexual activity measured by self-reported behaviors with that measured by healthcare claims for each person.

Using nationally representative survey data, linked with Medicaid administrative data, we were able to estimate the proportion of Medicaid women who were identified as sexually-active by self-reported behaviors and by healthcare claims, and to estimate chlamydia testing rates. The aim of this study was to understand sexual activity measures from different data sources and their impact on chlamydia testing rates when these measures were used as the denominator. In the future it should provide information for interventions to better monitor chlamydia testing for the young population in the United States.

## Methods

### NHANES Data

The National Health and Nutrition Examination Survey (NHANES), conducted by the National Center for Health Statistics (NCHS), is designed to assess the health and nutritional status of adults and children in the United States. The NHANES is unique in that it combines interviews and physical examinations. The interview includes demographic, socioeconomic, dietary, and health-related questions. Audio computer-assisted self-interviewing is used to ask sensitive questions, such as sexual behaviors, during the interview. The examination component consists of medical, dental, and physiological measurements, as well as laboratory tests administered by highly trained medical personnel. The sampling selection of the NHANES involves complex and multistage probability sampling design. A nationally representative sample of about 5,000 persons is surveyed each year for the civilian and non-institutionalized U.S. population. Response rates were 80% in 2001–2002 and 76% in 2003–2004 for women with both interviews and physical examinations.

### MAX Data

The Centers for Medicare & Medicaid Services (CMS) has created Medicaid Analytic eXtract (MAX) administrative data, which include enrollment and utilization data for persons from low income families who either enrolled in Medicaid or in the Children’s Health Insurance Program (CHIP) in all 50 states and the District of Columbia, for research and policy. The MAX enrollment file, known as the Person Summary (PS) file, includes enrollee-level information on enrollment status for each month, eligibility criteria for Medicaid or CHIP, and type of insurance plan (fee-for-service, or managed care). The MAX utilization data, which include inpatient files, prescription drugs files, long-term care files, and other services files, contain service utilization and date of services. The MAX PS files are person-level files, and the MAX utilization files are service-level files.

One of the ways to access the MAX data is through the CMS Virtual Research Data Center (VRDC). An Inter-Agency Agreement (IAA) for access to MAX data through VRDC was established between CDC and CMS in July 2013. Under the agreement, CDC researchers need to sign a data use agreement and submit their proposals to the CMS Privacy Board, which serves to ensure that patients’ privacy is protected and the need for identifiable data is justified. After the CMS Privacy Board approves the request, CDC researchers have access to a subset of the MAX data, according to the minimum data necessary rule.

### Data Linkage

NCHS and CMS have also created a set of analytic data files linking NHANES data to MAX data by NHANES respondent’s unique identifier.[18] The unique identifier includes Social Security number (SSN) and date of birth. The linked databases provide unique opportunities for research and policy analysis to study changes in health status, healthcare utilization, and cost in low income families with children, young adults, elderly adults, and disabled U.S. populations. CMS had provided NCHS with the MAX data for 1999 through 2007 for all successfully matched NHANES respondents. For example, the 2002 NHANES data with respondents who reported being enrolled in Medicaid in 2002 were matched with the MAX data by each year from 1999 through 2007, although many of these enrollees might not be enrolled in Medicaid for all years during 1999–2007. This linked dataset provides the opportunity to examine all healthcare utilization for the NHANES respondents who were matched with MAX data in the years they were enrolled in Medicaid during 1999–2007.

The linked analytic data with de-identified human participants generated from the linked data by NCHS are only accessible through the Research Data Center (RDC) at CDC after a research proposal is submitted and approved by the CDC review committee for purposes concerning potential disclosure risk.

### Data Used and Study Population

The 2001–2004 NHANES data were the most recent NHANES data available to be linked to the MAX data. The linked 2001–2004 NHANES-MAX data (2001–2004 NHANES data linked to 2000–2004 MAX data) were primarily used in this study. The study population included the 2001–2004 NHANES respondents who were women aged 18–25 years at interview, and matched to women in the 2000–2004 MAX data. The reason for using 2000–2004 MAX data rather than 2001–2004 MAX data was that enrollment and claims information in the 12 months prior to the survey interview was required for women surveyed in the 2001–2004 NHANES data. The reason that the study population included only women aged 18–25 years rather than women aged 15–25 years, used in the HEDIS measure for chlamydia testing, was that women aged ≥18 years in 2001–2004 NHANES data had been asked the number of their sexual partners during the past 12-month period. Women aged <18 years in the 2001–2004 NHANES data were not asked about their sexual behaviors in the past 12 months.

### Measures on Sexual Activity

#### Self-reported sexual activity

Of Medicaid women aged 18–25 years, their self-reported sexual activity was measured by two questions in the NHANES data, “Have you ever had sexual intercourse?” and, “In the past 12 months, with how many men have you had sexual intercourse?” Women aged 18–25 years were classified as sexually-active if they had sexual intercourse with one man in the past 12 months, and classified as non- sexually-active if they had never had sexual intercourse, or had no sexual intercourse in the past 12 months.

#### Claim-identified sexual activity

The claim-identified sexual activity was measured by claims for reproductive-related medical or prescription services among women who had ≥ one healthcare claim in the 12 months prior to their survey interview in the MAX data. We used the month of the survey interview as the index month, and considered the index month and 11 months prior to the index month as the 12 months prior to the survey interview. Reproductive health services were identified by their healthcare claims, using several standard coding systems—the International Classification of Disease, Ninth Revision, Clinical Modification (ICD-9-CM), the Physicians’ Current Procedural Terminology, 2012 (CPT 2012), National Drug Code Directory (NDC), and inpatient UB-92 revenue code (UB_92).[12] Reproductive-related claims were identified if women had, within the 12 months prior to the survey interview, healthcare claims that were associated with any of the following five reproductive health categories: 1) a cervical cancer screening service or pelvic examination, 2) a contraceptive service, 3) a pregnancy-related service, 4) an STD-related service, or 5) an infertility service. Women were classified as ineligible if they had no healthcare claim, or as eligible if they had healthcare claims in the 12 months prior to their survey interview. Of eligible women, they were classified as non-sexually-active if they had no reproductive-related claim, or as sexually-active if they had reproductive-related claims in the 12 months prior to their survey interview.

#### HEDIS-defined sexual activity

To be comparable to the denominator of the HEDIS measure on chlamydia testing (i.e., continuously enrolled without a gap of > one-month in Medicaid, and had ≥ one healthcare claim in the measurement year), HEDIS-defined sexual activity was measured by claims for reproductive-related medical or prescription services among women who had ≥one healthcare claim, and who were enrolled in Medicaid for ≥330 days in the 12 months prior to their survey interview. Women were classified as ineligible if they had no healthcare claim, or they were enrolled in Medicaid for <330 days, or as eligible if they had healthcare claims and they were enrolled in Medicaid for ≥330 days in the 12 months prior to their survey interview. Of eligible women, they were classified as non-sexually-active if they had no reproductive-related claim, or as sexually-active if they had reproductive-related claims in the 12 months prior to their survey interview.

### Chlamydia Testing and Additional Data Used

Chlamydia tests were identified by CPT codes if the tests were performed within the 12 months prior to the survey interview.[12]

Because the linked 2001–2004 NHANES-MAX data are more than 10 years from present, we also used the more recent data to confirm that the key findings from the linked 2001–2004 NHANES-MAX data are still relevant to the current healthcare environment. We used the 2011–2012 NHANES data, which are the most recent NHANES data available to the authors, and have not been linked to the MAX data by CMS and NCHS yet, to compare the proportion of Medicaid women aged 18–25 years who self-reported to be sexually-active between the linked 2001–2004 NHANES-MAX data and the 2011–2012 NHANES data. Similarly, we used the 2004 MAX data and the 2010 MAX data that are the most recent MAX data available to the authors through VRDC to compare the proportion of women aged 18–25 years who were enrolled in Medicaid for ≥330 days within the 12 months prior to the survey interview in the linked 2001–2004 NHANES-MAX data to that of women aged 18–25 years who were enrolled in Medicaid for ≥330 days in 2004, and in 2010, using the 2004 MAX data and the 2010 MAX data, respectively.

Because the 2004 and 2010 MAX data that the authors have access to through VRDC include only de-identified human participants, using 2004 and 2010 MAX data does not require a CDC institutional review board review.

### Statistical Analyses

Statistical analyses were conducted using the SAS System for Windows (release 9.3; SAS Institute Inc., Cary, NC) and SUDAAN (release 10.0; Research Triangle Institute, Research Triangle Park, NC). Most estimates incorporated the sample weight, and variance calculations accounted for the complex sample design of the NHANES data. All significance tests to compare weighted proportions were evaluated using a Chi-square test with two-sided p-value <0.05 as the level of statistical significance. Estimates with a sample size less than five counts were suppressed according to the NCHS guide. To demonstrate the number of days in Medicaid enrollment having impact on the proportion of women who had ≥ one healthcare claim or on chlamydia testing rate, we also estimated proportions of women who had ≥ one healthcare claim, and chlamydia testing rates between women with ≥330 days of enrollment and those with <210 days of enrollment. A simple analysis was conducted using SAS for the 2004 MAX data and the 2010 MAX data, because all Medicaid women aged 18–25 years–rather than a sample of them–were included.

## Results

Of annual 3.3 million Medicaid women aged 18–25 years weighted by a sample of 338 women in the linked 2001–2004 NHANES-MAX data, 91.5% (3.1 million women) self-reported to be sexually-active (Table 1). Self-reported sexual activity was not significantly associated with age, marital status, race/ethnicity, and the days of enrollment in the past 12 months. The comparison of three measures on sexual activity is presented in Table 2. Of 3.0 million and 1.8 million Medicaid women aged 18–25 years who were eligible for the claim-identified and the HEDIS-defined sexual activity measure, 73.3% and 69.8% were classified as sexually-active, respectively. Of 3.1 million women who self-reported to be sexually-active, 45.9% were ineligible for the HEDIS measure. Of HEDIS-defined non-sexually-active women aged 18–25, 77.5% self-reported to be sexually-active; of self-reported non-sexually-active women aged 18–25 years, 18.8% were HEDIS-defined sexually-active.

**Table 1:** Demographics and enrollment of linked Medicaid women aged 18–25 years (N = 338), 2001–2004.

	National Medicaid population	Self-reported sexual activity % (standard error)
Age group (year)		
18–19	757,522	88.0 (3.6)
20–25	2,578,380	92.5 (3.3)
Race/ethnicity		
Non-Hispanic white	1,455,297	90.9 (4.8)
Non-Hispanic black	1,102,390	93.7 (2.8)
Hispanic or other	778,215	89.3 (5.2)
Marital status		
Never married	2,001,541	87.9 (3.1)
Other	1,334,361	96.4 (3.6)
Days of enrollment		
1–209	757,534	96.2 (2.3)
210–329	667,715	93.5 (2.7)
≥330	1,910,653	88.8 (4.0)
Total	3,335,902	91.5 (2.7)

**Table 2:** Agreement of self-reported versus claim-identified versus HEDIS-defined sexual activity among annual 3.3 million Medicaid women aged 18–25 Years (N = 338), 2001–2004.

	Self-reported		Claim-identified			HEDIS-defined		
	Yes N (%)	No N (%)	Yes N (%)	No N (%)	Ineligible N (%)	Yes N (%)	No N (%)	Ineligible N (%)
Self-reported								
Yes (N = 3,050,918)	3,050,918 (100)	0 (0)	2,166,885 (71.0)	639,616 (21.0)	244,417 (8.0)	1,221,935 (40.1)	427,381 (14.0)	1,401,602 (45.9)
No (N = 284,984)	0 (0)	284,984 (100)	112,542 (39.5)†	172,442 (60.5)	†	53,657 (18.8)	123,862 (43.5)	107,465 (37.7)
Claim-identified								
Yes (N = 2,232,920)	2,166,885 (97.0)	66,035 (3.0)	2,232,920 (100)	0 (0)	0 (0)	1,275,592 (57.1)	0 (0)	957,328 (42.9)
No (N = 812,059)	639,616 (78.8)	172,443 (21.2)	0(0)	812,059 (100)	0 (0)	0 (0)	551,242 (67.9)	260,817 (32.1)
Ineligible (N = 290,923)	290,923 (100)‡	‡	0(0)	0(0)	290,923 (100)	0 (0)	0 (0)	290,923(100)
HEDIS-defined								
Yes (N = 1,275,592)	1,221,935 (95.8)	53,657 (4.2)	1,275,592 (100)	0 (0)	0 (0)	1,275,592 (100)	0 (0)	0 (0)
No (N = 551,243)	427,382 (77.5)	123,861 (22.5)	0 (0)	551,243 (100)	0 (0)	0 (0)	551,243 (100)	0 (0)
Ineligible (N = 1,509,067)	1,401,602 (92.9)	107,465 (7.1)	957,328 (63.4)	260,817 (17.3)	290,922 (19.3)	0 (0)	0 (0)	1,509,067 (100)

† Estimate was combined from ‘Yes’ category and ‘Ineligible’ category.

‡ Estimate was combined from ‘Yes’ category and ‘No’ category.

**Fig 1:**
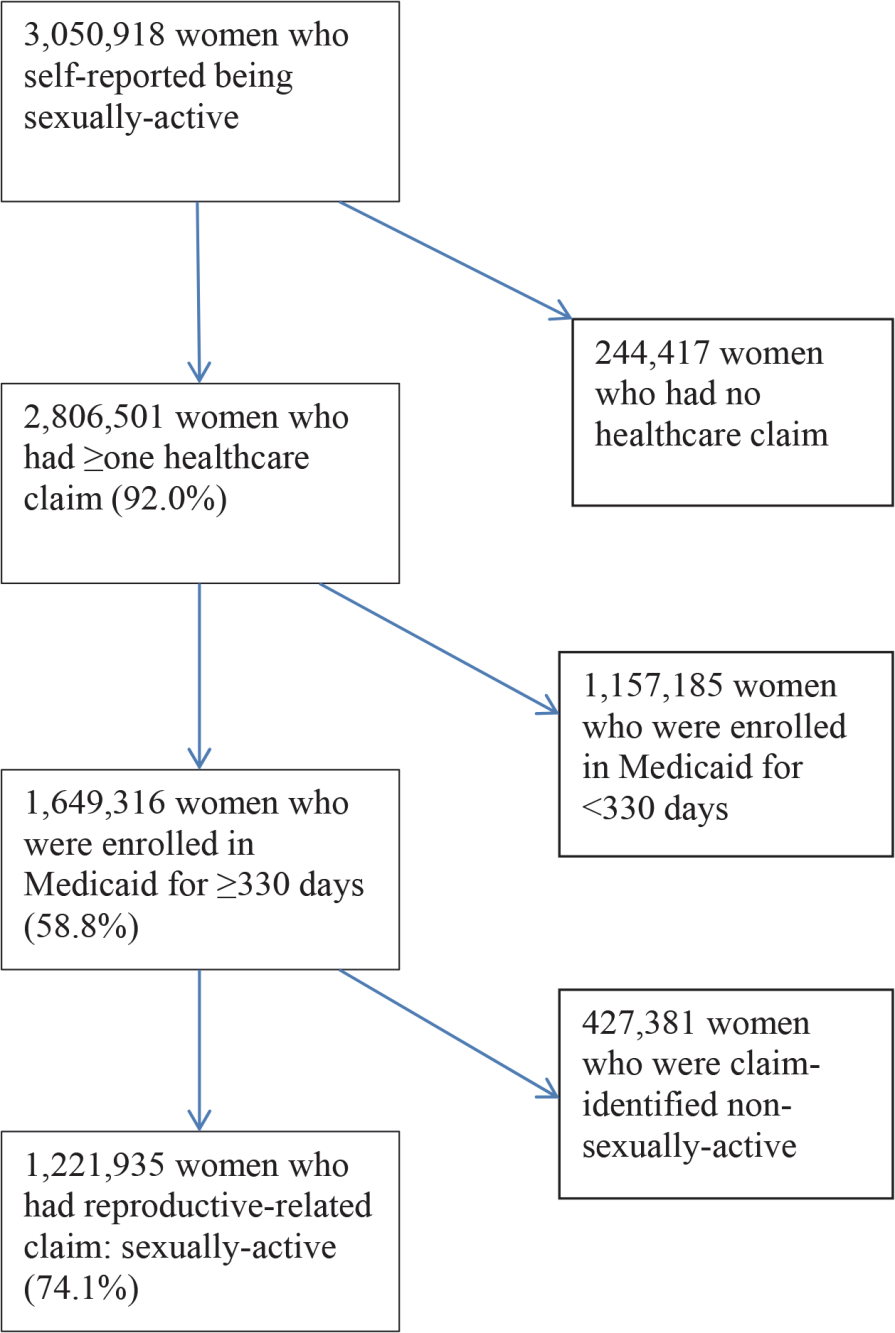
Steps were used in identifying the number of HEDIS-defined sexually-active women among self-reported sexually-active women.

Of 3.1 million women who self-reported to be sexually-active, 40.1% were sexually-active by the HEDIS-defined measure (Fig 1). More specifically, of these 3.1 million women, 92.0% had ≥ one healthcare claim; of the self-reported sexually-active women who had ≥ one healthcare claim, 58.8% had been enrolled in Medicaid for ≥330 days in the 12 months prior to the survey interview; of these self-reported sexually-active women with ≥330 days of Medicaid enrollment, 74.1% had healthcare claims to indicate that they were sexually-active.

Using the HEDIS measure, 42.4% of the HEDIS-defined sexually-active women aged 18–25 years had chlamydia testing (Table 3). Chlamydia testing rate was not significantly associated with age, marital status, and race/ethnicity among the HEDIS-defined sexually-active women. Of self-reported sexually-active women aged 18–25 years, 30.7% (SE = 5.0) had chlamydia testing. Of self-reported sexually-active women aged 18–25 years with ≥330 days of Medicaid coverage who had ≥ one healthcare claim, 32.6% (SE = 8.3%) had chlamydia testing. Of women aged 18–25 years who were identified to be sexually-active by both the self-reported measure and by the HEDIS-defined measure, 44.0% (SE = 9.7%) had chlamydia testing.

**Table 3:** HEDIS-defined sexual activity and chlamydia testing rates among women aged 18–25 years who were eligible for the HEDIS measure (N = 172), 2001–2004.

	Population	HEDIS-defined sexual activity % (standard error)	Chlamydia testing rate% (standard error)
Age groups (years)			
18–19	355,093	58.8 (8.8)	49.1 (10.5)
20–25	1,471,742	72.5 (6.3)	41.1 (10.5)
Race/ethnicity			
Non-Hispanic white	682,616	67.8 (10.3)	26.1 (11.4)
Non-Hispanic black	714,950	63.9 (10.0)	45.0 (8.2)
Hispanic or other	429,269	83.0 (8.0)	60.3 (14.2)
Marital status			
Never married	1,094,297	63.9 (7.9)	29.9 (8.0)
Other	732,538	78.7 (7.5)	57.5 (12.6)
Total	1,826,835	69.8 (5.7)	42.4 (9.7)

Of self-reported sexually-active women aged 18–25 years, women who had enrolled in Medicaid for ≥330 days were more likely to have ≥ one healthcare claim than those who had enrolled for < 210 days (95.5% SE = 2.2% versus 81.0% SE = 7.6%). Of claim-identified sexually-active women aged 18–25 years, the chlamydial testing rate decreased from 42.4% (SE = 9.7) among women with ≥330 days of enrollment to 24.9% (SE = 7.1%) among women with <210 days of enrollment.

Of Medicaid women aged 18–25 years, 57.3% were enrolled in Medicaid for ≥330 days in the linked 2001–2004 NHANES-MAX data versus 50.9% in the 2004 MAX data, versus 53.9% in the 2010 MAX data. Of Medicaid women aged 18–25 years, 91.5% self-reported to be sexually-active in the linked 2001–2004 NHANES-MAX data, versus 92.9% in the 2011–2012 NHANES data.

## Discussion

To the best of our knowledge, this is the first nationally representative study to use both self-reported survey data and healthcare utilization data to research women’s sexual activity. Comparing self-reported survey data to healthcare utilization data, our findings showed that many of the self-reported sexually-active women aged 18–25 years had no healthcare utilization record, were not continuously enrolled in Medicaid, or had no reproductive-related healthcare utilization in the 12 months prior to the survey interview. Because many of self-reported sexually-active women were ineligible for HEDIS-defined measure during 2001–2004, as a result, only about 40% of self-reported sexually-active women were the HEDIS-defined sexually-active women in the Medicaid population, a denominator in the HEDIS measure on chlamydia testing. With consistent and low proportions of Medicaid women aged 18–25 years who were enrolled for ≥330 days in Medicaid, 50.9% in the 2004 MAX data versus 53.9% in the 2010 MAX data versus 57.3% in the linked 2001–2004 NHANES-MAX data for Medicaid women, our study highlighted that the denominator used in the HEDIS measure on chlamydia testing had limited representation of Medicaid women aged 18–25 years under the current healthcare environment.

There is a limitation of measuring quality of healthcare in Medicaid when a high proportion of women were excluded from the measure. Several administrative changes for improving continuity of Medicaid coverage, such as changes in renewal policy or extended eligibility criteria to young adults, have been suggested in previous studies.[19,20] With more and more states implementing these policies, Medicaid’s continuity of coverage will improve. Consequently, healthcare quality measures will be more representative, because most eligible Medicaid people will be included in the HEDIS measures.

Our chlamydia testing rates also varied according to the denominator measures used. The testing rate was approximately 31% for all self-reported sexually-active women, but 44% among women who were identified as sexually-active by both the self-reported measure and by the HEDIS-defined measure. Our HEDIS measure on chlamydia testing rate was similar to that reported using NCQA data.[21] Our findings suggest that the measure and the data sources need to be stated clearly when chlamydia testing rates are reported. Our findings reconfirmed that for results to be similar to the HEDIS measure on chlamydia testing, women aged 15–25 years should be limited to those who were continuously enrolled in health plans for ≥ 330 days, and those who had reproductive-related claims during the measurement year.

The results of this study, plus the results from a recent related study, can provide information to better understand on issues related to the measures for sexual activity.[17] Several estimates from this study were within a reasonable range compared to the recent related study. For example, the proportion of women who were HEDIS-defined sexually-active among self-reported sexually-active women ranged from 74% in this study to 85% in the recent related study; the chlamydia testing rate ranged from 42% in this study to 40% in the recent related study. In addition, many associations related to sexual activity were similar between these two studies. Although there are several differences related to study design between these two studies, such as years of the data used, Medicaid patients versus privately-insured patients, the nationally representative data versus health plan data, the use of audio computer-assisted self-interviewing versus the mail survey for confidentiality concerns, and a high response rate in this study versus a low response rate in the recent related study, the two studies are complementary to each other. The unique contributions of these two studies are that our study highlights that the denominator used in the current HEDIS measure on chlamydia testing has limited representation of Medicaid women, mainly due to insurance coverage, and the other study showed that for women aged 18–25 years who self-reported non-sexually-active, approximately 64% were classified as non-sexually-active by the HEDIS measure.

This study had a number of strengths. The NHANES data had a high response rate. It collected valuable, sensitive data using audio computer-assisted self-interviewing to reduce participant’s confidentiality concerns. The NHANES data provided national estimates of sexual activity for Medicaid women. The estimates of sexual activity in the NHANES data were consistent from 2001 to 2012 in this study. The linked NHANES-MAX data offered valuable information for public health research. Self-reported sexual behaviors are much better than medical records or healthcare claims to identify sexual activity, while healthcare claims may be much better than self-reports to identify the procedure of chlamydia testing. There were, however, several limitations in this study. First, the sample used represented only the Medicaid population, rather than the general population. Second, our study excluded women aged <18 years due to lack of self-reported sexual behaviors. Estimates among women aged 15–17 years may differ from the ones presented in this study. Third, with a limited sample size for non-sexually-active women classified by the self-reported measure, we could not do any further analysis by age, race/ethnicity, or marital status. Fourth, sexually-active women aged 18–25 years and chlamydia testing rate might be underestimated, because 17% of procedure codes were under other coding systems that could not be used to correctly identify sexuality or chlamydia testing in the MAX data. Fifth, although our data showed that several key findings from the linked 2001–2004 NHANES-MAX data are relevant to the current healthcare environment, the linked data are more than 10 years from present. Finally, some women might not have been identified as sexually-active, or having chlamydia testing, if they obtained out-of-plan care for their reproductive-related service, and chlamydia testing that did not generate claims in the MAX data.

Our study suggests that the number of sexually-active women aged 18–25 years used as a denominator in chlamydia testing could be significantly different, depending on the measures applied and the data used. Therefore, when a chlamydia testing rate is reported, it is necessary to describe clearly the measure and data sources used. Although the HEDIS measure provides a consistent measure with which to track trends over time, our study highlighted key areas where the HEDIS measure is suboptimal. Our data especially highlighted the limited representativeness of Medicaid population in measuring quality of healthcare when a high proportion of Medicaid population is enrolled in Medicaid for <330 days.
